# Geographical structuring and low diversity of paternal lineages in Bahrain shown by analysis of 27 Y-STRs

**DOI:** 10.1007/s00438-020-01696-4

**Published:** 2020-06-25

**Authors:** Noora R. Al-Snan, Safia A. Messaoudi, Yahya M. Khubrani, Jon H. Wetton, Mark A. Jobling, Moiz Bakhiet

**Affiliations:** 1 Department of Molecular Medicine, College of Medical and Medicine Sciences, Arabian Gulf University, Manama, Kingdom of Bahrain; 2 Forensic Science Laboratory, Directorate of Forensic Science, General Directorate of Criminal Investigation and Forensic Science, Ministry of Interior, Manama, Kingdom of Bahrain; 3 Forensic Sciences Department, College of Criminal Justice, Naif Arab University for Security Sciences, Riyadh, Saudi Arabia; 4 Department of Genetics and Genome Biology, University of Leicester, University Road, Leicester, UK; 5 Forensic Genetics Laboratory, General Administration of Criminal Evidence, Public Security, Ministry of Interior, Riyadh, Saudi Arabia

**Keywords:** Bahrain, Y-STRs, Haplogroup, Population structure, Haplotype

## Abstract

We have determined the distribution of Y-chromosomal haplotypes and predicted haplogroups in the ethnically diverse Kingdom of Bahrain, a small archipelago in the Arabian Gulf. Paternal population structure within Bahrain was investigated using the 27 Y-STRs (short tandem repeats) in the Yfiler Plus kit to generate haplotypes from 562 unrelated Bahraini males, sub-divided into four geographical regions—Northern, Capital, Southern and Muharraq. Yfiler Plus provided a significant improvement over the 17-locus Yfiler kit in discrimination capacity (from 77% to 87.5% overall), but discrimination capacity differed widely between regions from 98.4% in Muharraq to 75.2% in the Northern region, an unusually low value possibly resulting from recent rapid population expansion. Clusters of closely related male lineages were seen, with only 79.4% of donors displaying unique haplotypes and 59% of instances of shared haplotypes occurring within, rather than between, regions. Haplogroup prediction indicated diverse origins of the population with a predominance of haplogroups J2 and J1, both typical of the Arabian Peninsula, but also haplogroups such as B2 and E1b1a likely originating in Africa, and H, L and R2 likely indicative of migration from South Asia. Haplogroup frequencies differed significantly between regions, with J2 significantly more common in the Northern region compared with the Southern, possibly due to differential settlement by Baharna and Arabs. Our study shows that paternal lineage population structure can exist even over small geographical scales, and that highly discriminating genetic tools are required where rapid expansions have occurred within tightly bounded populations.

**Electronic supplementary material:**

The online version of this article (10.1007/s00438-020-01696-4) contains supplementary material, which is available to authorized users.

## Introduction

**Fig. 1:**
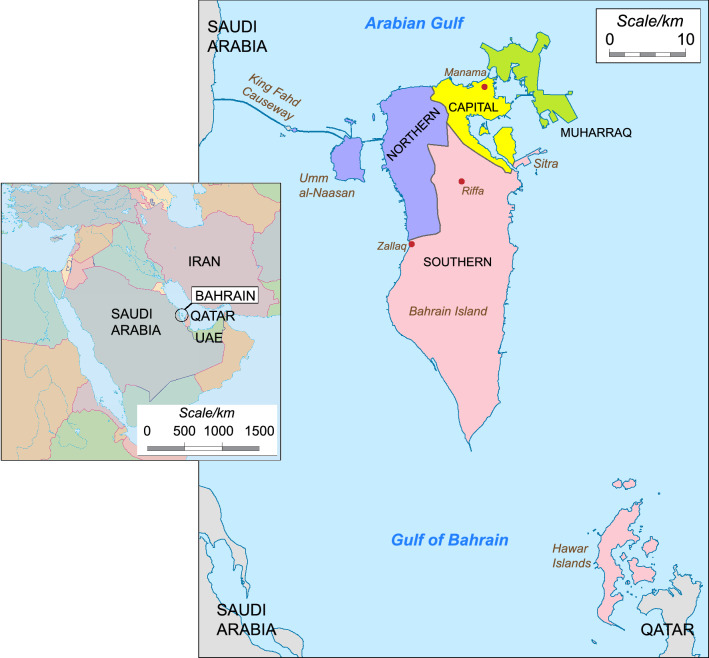
Map of Bahrain. The main map shows the location of places mentioned in the text, and the four governorates used as a basis for sample subdivision. The inset map to the left shows the position of Bahrain in its regional context. UAE: United Arab Emirates. Main map adapted from commons.wikimedia.org/wiki/File:Bahrain,_administrative_divisions_-_de_-_colored.svg (GNU Free Documentation License: commons.wikimedia.org/wiki/Commons:GNU_Free_Documentation_License,_version_1.2). Inset map from Mountain High Map Frontiers™ version 94.01 (Mountain High Maps® Copyright © 1993 Digital Wisdom®, Inc.; www.digiwis.com/dwi_frl.htm)

The Kingdom of Bahrain is an archipelago totaling just 765 square kilometers, located northwest of the State of Qatar, and east of the Kingdom of Saudi Arabia; further to the north and east lies the Islamic Republic of Iran (Abdulla and Zain al Abdeen 2009) (see Fig. 1). Bahrain’s population stood at ~ 1.6 million in 2019, of which less than half are Bahraini citizens (www.worldometers.info)**,** distributed primarily on the main islands of Bahrain, Muharraq, Umm al-Naasan and Sitra (Hitti and Murgotten 1916). Its coastal location in the Arabian Gulf, fertile land and abundance of fresh water have attracted many migrants resulting in an ethnically diverse society with origins both in the Arabian Peninsula and places further afield such as Iran and India (Holes 2001). Bahrain's pre-Islamic population consisted of Christian Arabs, Persians (Zoroastrians), Jews, and Aramaic-speaking agriculturalists (Lawson 1989) and now comprises four main ethnic groups: Arabs, Baharna (the putative indigenous peoples) and Persians (Huwala and Ajam) which are distributed unevenly between the four Governorates (Capital, Muharraq, Northern and Southern) (al-Khūrī 1980; Fuccaro 2009). The Arabs have traditionally lived in areas such as Zallaq, Hawar Islands, Riffa (all in the Southern Governorate) and Muharraq. The Ajam, who are ethnic Persians (McCoy 2008), and Baharna, the Arabized descendants of the pre-Islamic population, form large communities in the Capital Governorate, Muharraq and in some parts of the Northern Governorate. Muharraq and Riffa also have significant numbers of Huwala, descendants of migrant Arabs many of whom had journeyed from the Arabian Peninsula to Iran in the eighteenth or nineteenth centuries, largely returning between 1850 and 1900, as well as Bahrainis of Balochi descent and others of East African origin (Lawson 1989). This geographical and social organization might be expected to have an effect on patterns of genetic diversity, particularly considering the male-specific region of the Y chromosome (MSY), which tends to show pronounced geographic variation (de Knijff et al. 1997; Roewer 2009; Ballantyne and Kayser 2012). To date, genetic studies of the Bahraini population have been limited and little has been done to characterize population structure within the Kingdom.

Here we use the Yfiler Plus kit to characterize Y-STR (short tandem repeat) haplotypes in 562 unrelated male Bahraini citizens sub-divided by geographical region; the same samples have been previously typed with autosomal STRs (Al-Snan et al. 2019). The 6-dye Yfiler Plus PCR Amplification kit detects 27 Y-STR loci: DYS19, DYS385a/b, DYS389I/II, DYS390, DYS391, DYS392, DYS393, DYS437, DYS438, DYS439, DYS448, DYS456, DYS458, DYS460, DYS481, DYS533, DYS635 (Y-GATA-C4), Y-GATA-H4, DYF387S1a/b, DYS449, DYS518, DYS570, DYS576 and DYS627, with the last seven loci being ‘rapidly mutating Y-STRs’ (RM Y-STRs). These RM Y-STRs, which were not detected by the original Yfiler multiplex, are particularly useful for distinguishing between closely related males (Ballantyne et al. 2010, 2014; Adnan et al. 2016) and increase the likelihood of discrimination in populations that have grown very rapidly often from small numbers of patrilineally-related tribal groups (Khubrani et al. 2018).

## Materials and methods

### Sample collection

Blood spots were collected on Nucleic-Cards (Copan, Italy) from 562 unrelated Bahraini males whose ancestry to the level of paternal grandfather was assigned to one of four administrative subdivisions of the country (Capital, Muharraq, Northern and Southern Governorates). Donors ranging in age from 20 to 55 years were recruited through social media channels such as Twitter and Instagram and invited to the General Directorate of Criminal Investigation and Forensic Science—Kingdom of Bahrain to submit blood samples. Ethical review for the study was provided by the Research and Research Ethics Committee (RREC) (E007-PI-10/17) in the Arabian Gulf University, and informed consent was provided by all participants along with details sufficient to exclude individuals sharing ancestry closer than paternal great grandfather.

### DNA amplification and fragment detection

DNA was obtained from 1.2-mm diameter discs punched from blood-spots using the easyPunch STARlet system (Hamilton). A total of 27 Y-STRs were directly amplified with the Yfiler Plus Amplification Kit using 28 PCR cycles according to manufacturer’s recommendations on a Veriti 96-well Thermal Cycler (Thermo Fisher Scientific). The PCR products (1 µl) were separated by capillary electrophoresis in an ABI 3500xl Genetic Analyzer along with LIZ600 size standard v2 in a Hi-Di Formamide master mix (all Thermo Fisher Scientific). GeneMapper ID-X Software v1.4 was used for genotype assignment using the allelic ladders provided with the Yfiler Plus kit following ISFG recommendations (Gusmão et al. 2017). Samples displaying non-standard patterns, off-ladder and microvariant alleles were repeated. The resulting profiles were submitted to the Y-chromosomal Haplotype Reference Database (YHRD) (Roewer 2017) under the following accession numbers: Capital Governorate YA004556, Northern Governorate YA004557, Southern Governorate YA004558 and Muharraq Governorate YA004559.

### Statistical analysis

#### Forensic and population genetic parameters

Haplotype information (number of haplotypes, number of unique haplotypes, discriminatory capacity and haplotype diversity) was calculated using STRAF (Gouy and Zieger 2017). Genetic diversity (GD) was calculated according to Nei and Tajima (1981), haplotype match probability (HMP) was calculated as the sum of the squared haplotype frequencies, and the discriminatory capacity (DC) was calculated as the ratio between the number of different haplotypes and the total number of haplotypes. Haplotype diversities (HD) were calculated as one minus the HMP multiplied by the number of haplotypes, divided by the number of haplotypes minus one.

Genetic distances between populations were evaluated using the *R*_ST_ statistic, and visualized through multi-dimensional scaling (MDS) plots using comparative population data and the calculation tool within the online YHRD (Willuweit and Roewer 2015). Population differentiation tests based on predicted haplogroup frequencies were carried out within Arlequin (Excoffier and Lischer 2010).

For all statistical analyses, the repeat number of DYS389I was subtracted from that of DYS389II so that its diversity was not considered twice.

#### Haplogroup prediction

The NevGen Y-DNA Haplogroup Predictor (Ćetković and Nevski 2016), based on a previously-implemented Bayesian approach (Athey 2006), was used to derive likely Y-SNP haplogroups from Y-STR haplotypes (Ćetković and Nevski 2016). The software bases the prediction upon length variation at the 23 loci in the Promega PowerPlex Y23 multiplex, so DYS549 and DYS643 (which are not amplified by Yfiler Plus) were coded as missing data. While the Predictor allocates haplotypes to one of 484 sub-branches of the Y haplogroup tree, we have restricted our level of assignments to major haplogroups which we have previously shown can be accurately predicted in Arabian Peninsula populations (Karafet et al. 2008; Khubrani et al. 2018).

#### Median-joining networks

Median-joining networks of haplotypes were constructed using the program Network v. 5.0.1.1 (Bandelt et al. 1999) with weightings based on the inverse variance of repeat length at each locus in order to reduce reticulation. The duplicated loci DYS385a/b and DYF387S1a/b were excluded from the network construction as it is not possible to associate particular alleles to specific copies.

## Results

### Haplogroup prediction and median-joining networks

**Fig. 2:**
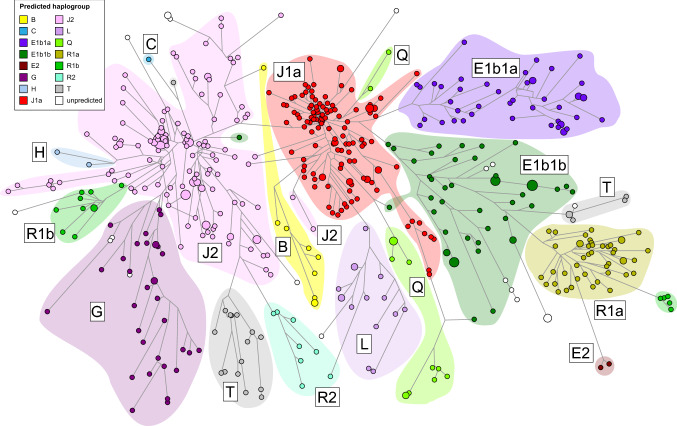
Median-joining network based upon Yfiler Plus haplotypes from 562 Bahraini males showing concordance of NevGen haplogroup predictions with the branching structure. Nodes represent haplotypes, with node size proportional to the number of men sharing the haplotype. Lines connecting nodes have lengths proportional to the number of mutational steps with the shortest line corresponding to a difference of one repeat unit at a single locus. Each node is colored according to its NevGen haplogroup prediction as shown in the key, including 20 haplotypes having a probability of inappropriate prediction greater than 97.5% which are shown as white, unpredicted (UP) nodes. Shapes enclose groups of haplotypes predicted to belong to the same major haplogroup as indicated by the adjacent labels

A median-joining network based solely on allele length differences among the haplotypes of the 562 Bahraini males displays several distinct branches as shown in Fig. 2. An almost perfect correspondence was noted between these and the haplogroup predictions. Haplogroup prediction suggests that haplogroup J2 is the most common in the Bahraini population encompassing 27.6% of the sample, followed by J1 (23.0%), E1b1b (8.9%), E1b1a (8.6%) and R1a (8.4%), with other predicted haplogroups (G, T, L, R1b, Q, R2, B2, E2, H and C) occurring at progressively lower frequencies. The profiles of 20 men were assigned a low haplogroup fitness (< 25) by the NevGen software, and also received a high probability of haplogroup misassignment (> 97.5%) because there were no closely matching haplotypes of known haplogroup within the NevGen prediction model; these were therefore designated as unpredicted (UP).

**Fig. 3:**
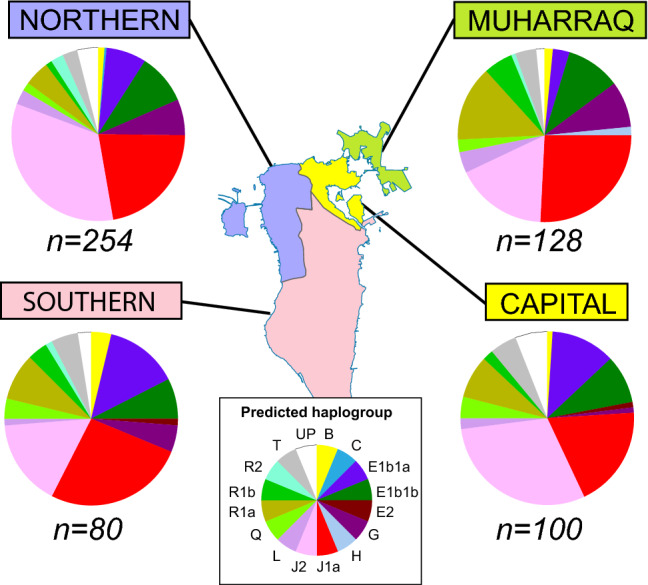
Proportions of predicted haplogroups observed in the four governorates of Bahrain. Sector area in the pie-charts indicates the frequency of predicted haplogroups, as shown by colors in the key below. Sample sizes (*n*) for each governorate are also given

Haplogroup predictions allow comparison with SNP-defined lineages that are known from published studies to differ significantly in frequency between countries. Table 1 and Fig. 3 and Online Resource 1 demonstrate that haplogroup frequency differences also exist between governorates. The ratio of haplogroup J1 to J2 shows clear sub-population differences (Fisher’s Exact Test *P* = 0.012). Haplogroup J1 is most frequent in the Southern Governorate (27%) where the highest proportion of Arabs live, and in the Muharraq Governorate (27%) where many migrant Huwala Arabs resettled, and it declines to its lowest frequency in the Northern and Capital Governorates (21% and 19%). By contrast, the Northern and Capital Governorates where the Baharna and Ajam are most represented show higher frequencies of haplogroup J2 (34% and 31%) than in Muharraq and the Southern Governorate (both 17%).

**Table 1:** Sub-regional affiliations of predicted Y-haplogroups for 562 Bahraini males

Haplogroup	*n*	B	C	E1b1a	E1b1b	E2	G	H	J1	J2	L	Q	R1a	R1b	R2	T	UP
Capital	**100**	1		12	9	1	1		19	30	2	4	8	2		5	6
Muharraq	**128**	2		4	13		11	2	33	22	5	3	18	7	1	5	2
Northern	**254**	3	1	19	24		17		56	85	7	4	12	3	6	7	10
Southern	**80**	3		11	6	1	4		21	13	1	3	7	3	1	4	2
Bahrain	**562**	9	1	46	52	2	33	2	129	150	15	14	45	15	8	21	20

Number of individuals are shown in bold

*n* number of individuals, *UP* unpredicted haplogroup

Pairwise F_ST_ based on predicted haplogroup frequencies shows that the greatest overall differences are between Muharraq and Northern Governorates (*F*_ST_ = 0.01906, *P* < 0.0001) and Muharraq and Capital Governorates (*F*_ST_ = 0.01586, *P* < 0.0001) with other comparisons not reaching formal significance after Bonferroni correction (Table 2). Similarly, Fisher’s Exact Tests of population differentiation highlighted the same pairs as being significant with the comparison between the Northern and Southern Governorates once again failing to reach significance after Bonferroni correction. A similar pattern was seen when considering *R*_ST_ based on the Y-STR haplotypes themselves but in this case while Muharraq and Northern Governorates (*R*_ST_ = 0.01179, *P* < 0.0001) remained highly significant, the other comparisons were not significant following Bonferroni correction (Table 3).

**Table 2:** *F*_ST_ values for pairwise comparisons of predicted haplogroup frequency

	Capital	Northern	Muharraq	Southern
Capital	0	0.3603 ± 0.058	** < 0.00001**	0.1441 ± 0.034
Northern	0	0	** < 0.00001**	0.0180 ± 0.012
Muharraq	0.01586	0.01906	0	0.3784 ± 0.030
Southern	0.00555	0.01599	0.00051	0

*F*_ST_ values for pairwise comparisons of predicted haplogroup frequency shown below the diagonal, and probabilities shown above; those which were significant after Bonferroni correction are shown in bold

**Table 3:** *R*_ST_ values for pairwise comparisons of haplotypes between populations

	Capital	Northern	Muharraq	Southern
Capital	0	0.0811 ± 0.037	0.0451 ± 0.020	0.3333 ± 0.051
Northern	0.00404	0	** < 0.00001**	0.0180 ± 0.012
Muharraq	0.00736	0.01179	0	0.2162 ± 0.043
Southern	0.00211	0.00894	0.00182	0

*R*_ST_ values for pairwise comparisons of haplotypes between populations shown below the diagonal, and probabilities shown above; those which were significant after Bonferroni correction are shown in bold

### Y-STR allele and haplotype diversity

Microvariant alleles aligning with the Yfiler Plus virtual allelic ladder were detected in 141 samples; 129 samples displayed ‘.2’ microvariants at DYS458 (14.2, 16.2, 17.2, 18.2, 19.2, 20.2, 21.2 and 22.2), which corresponded exactly with haplotypes predicted to belong to haplogroup J1 in concordance with the well-established association between intermediate length (.2) DYS458 alleles and this haplogroup (Ferri et al. 2008; Khubrani et al. 2018). Four samples displayed .2 microvariants present in the DYF387S1 virtual ladder (39.2 and 41.2), and all were predicted to belong to haplogroup B2 which is often associated with such microvariants at this locus (Iacovacci et al. 2017). Similarly, a DYS481 25.1 microvariant allele in five men was wholly concordant with the NevGen prediction of the R1b-PH155 subclade, which is a globally rare basal R1b lineage previously recorded in several Bahrainis who have very similar haplotypes to those typed here, and have submitted their Y-STR and Y-SNP profiles to the YFull Tree database (Roewer 2017; Xie et al. 2019). The same allele (25.1) has also been recorded in a Han Chinese individual who was derived for the Y-SNP M343 but ancestral for SNP L389, which also implies a basal R1b lineage (Guo 2017).

Several off-ladder (OL) alleles were designated after comparison with the allelic ladder. Six OL alleles were detected at DYF387S1; a single example of 36.3 in a predicted G2a haplotype, 35.2 in two individuals sharing an identical haplotype predicted to belong to haplogroup R1a, and a single instance of 44.2 and two occurrences of 42.2 in three different predicted B2a haplotypes (these representing OL variants of the previously mentioned association of .2 alleles with the B2 haplogroup). Two occurrences of the OL DYS570 allele 14.3 were found in closely related haplotypes predicted to belong to haplogroup J1a, and differing by a single repeat at each of four loci. Three OL alleles were detected at DYS449 (two instances of 34.2 and a single 32.1) all within closely-related predicted E1b1 lineages. The clustering of intermediate alleles is shown in the median-joining network in Fig. S2.

An unusual finding was an OL peak of 219 bp within the DYS19 size range in association with a standard DYS19 allele 15. This donor lacked a peak within the expected size range of DYS448 which shares the same dye, raising the possibility that the OL peak is the result of an unusually large internal deletion producing a DYS448 allele 4. The donor was predicted to belong to haplogroup J2a, which typically has alleles in the range of 19–23 repeats (309–334 bp). The common structure of alleles at this locus comprises two variable length (AGAGAT)_n_ hexamer repeats separated by a 42-bp “non-variable” region of seven diverged hexamer repeats; the observed OL peak is appropriately sized to have resulted from the loss of between 15 and 19 hexamer repeats. A similar event has been reported previously by Budowle et al. (Budowle et al. 2008). As well as this “pseudo-null” DYS448 allele, a true null allele was detected at DYS439 in a donor of unpredicted haplogroup.

Duplications at DYF387S1 produced two instances of tri-allelic patterns similar to those reported in several populations (Ye et al. 2015; Pickrahn et al. 2016; Wang et al. 2016; Iacovacci et al. 2017; Spólnicka et al. 2017; Watahiki et al. 2019). In one predicted haplogroup J1a male the alleles 36, 37 and 38 were all detected with equal strength implying an equal copy number, as would be seen if just one DYF387S1 copy had been duplicated. A second donor who was predicted as haplogroup E1b1b-V13 displayed alleles 35, 36 and 37, but the allele 35 peak was double the height of the others, implying that there were four copies of the locus in this individual. The different haplogroup affiliations show that duplications in this region are recurrent, and have varied consequences.

Another possible mutational event associated with duplicated STR loci is gene conversion leading to the homogenization of the repeat number between two copies (Balaresque et al. 2014). This process may explain why two predicted haplogroup B2 individuals share a Y-STR haplotype containing only a 37-repeat DYF387S1 peak. The other seven predicted B2 haplotypes in this study all display one copy of DYF387S1, with an integer number of repeats differing by at least three repeats from an intermediate (.2) copy as is frequently observed in other B2 lineages (Iacovacci et al. 2017). It is likely there was a gene conversion back to the ancestral (integer) state earlier in the paternal lineage of these two men.

The complete haplotype list and diversity summary statistics are provided for the four sub-populations (Online Resource 1 and Table 4, respectively). As expected, the number of haplotypes was considerably increased by the ten additional loci compared with the earlier Yfiler multiplex; 492 distinct Yfiler Plus haplotypes were detected in the 562 samples. Of these, 446 haplotypes were observed once (90.7%), and there were 33 identical pairs, eight trios, three quartets, one quintet and one haplotype shared nine times.

**Table 4:** Diversity summary statistics for Y-STR haplotypes, considering region of recruitment

Population	n*	No. unique hts	No. pair hts	No. trio hts	4	5	6	9	13	19	HMP	Haplotype Diversity	% unique hts	DC
Yfiler Plus Amplification Kit														
All	**562**	446	33	8	3	1		1			0.0025	0.9992	79.4	87.5
Capital	**100**	90	5								0.0110	0.9990	90.0	95.0
Muharraq	**128**	124	2								0.0081	0.9998	96.9	98.4
Northern	**254**	191	16	4	2	1	1				0.0060	0.9980	75.2	84.6
Southern	**80**	71	3	1							0.0144	0.9981	88.8	93.8
Yfiler loci only														
All	**562**	359	49	17	5	3				1	0.0039	0.9979	63.9	77.0
Capital	**100**	78	6	2	1						0.0136	0.9964	78.0	87.0
Muharraq	**128**	108	10								0.0090	0.9988	84.4	92.2
Northern	**254**	165	17	8	2	2			1		0.0087	0.9952	65.0	76.4
Southern	**80**	67	5	1							0.0150	0.9975	83.8	91.3

Number of individuals are shown in bold

*n number of individuals, *hts* haplotypes, *HMP* haplotype match probability, *DC* discrimination capacity. Yfiler comparison: lists statistics considering only those STRs included in the 17-STR Yfiler kit

Considering all the Yfiler Plus loci the proportion of donors within the national sample with unique haplotypes was 79.4%, declining to 63.7% when only the Yfiler loci were analyzed. The diverse Muharraq Governorate had the highest percentage of unique haplotypes (96.9% for Yfiler Plus, reducing to 84.4% for Yfiler), while the lowest percentage bearing unique haplotypes was seen in the Northern Governorate (75.2% reducing to 64.6% at the Yfiler loci). The majority of shared haplotypes were shared within rather than between regions (59% against an expectation of 31% if lineages were randomly dispersed across the governorates) and clusters of related haplotypes also appear more frequently within rather than across governorates (see Fig. S1).

This was naturally reflected in the discriminatory capacity (DC), where the national value for the Yfiler Plus loci was 87.5%, considerably higher than when considering Yfiler (77.0%). Regarding the subpopulations, Muharraq Governorate recorded the highest DC (98.4%) followed by Capital Governorate (95.0%), with Northern Governorate the lowest at 84.6%.

### MDS analysis

**Fig. 4:**
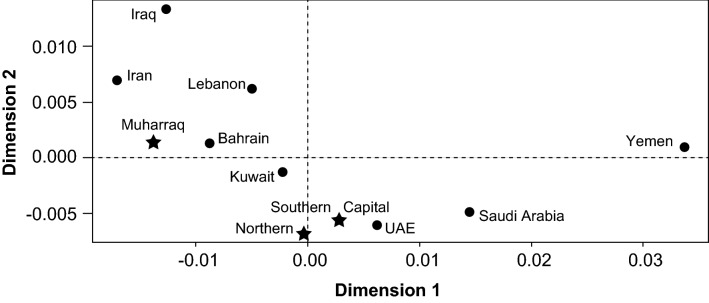
Multidimensional scaling (MDS) plot showing relationships between Bahraini and other populations. Comparison with other datasets required reduction of the number of STRs to a shared set of seventeen (Yfiler loci). Comparison is shown of the four governorate samples from the current study (stars) with an independent dataset of 156 Y-STR haplotypes from the same country (‘Bahrain’ (YHRD data)), and other datasets from the region: Saudi Arabia (*n* = 597 (Khubrani et al. 2018)), Iraq (*n* = 1354 (Purps et al. 2014)), Iran (*n* = 2333 (Nasidze et al. 2003; Alshamali et al. 2009; Roewer et al. 2009)); Kuwait (*n* = 534 (Triki-Fendri et al. 2010; Taqi et al. 2015)), UAE (*n* = 809 (Nazir et al. 2016)), Yemen (n = 149 (YHRD data)) and Lebanon (*n* = 555 (YHRD data))

AMOVA was used to explore population differentiation with pairwise genetic distances (*R*_ST_) visualized by multidimensional scaling (MDS), between the four Governorates of Bahrain and relevant neighboring population samples obtained from YHRD Release R61 (Fig. 4). Comparisons were performed using only the Yfiler loci, as Yfiler Plus data were not available for Iran, the region from which a significant proportion of the Bahraini population originates. Muharraq shows a greater degree of similarity to Iran whereas Southern, Northern and Capital populations were closer to Emirati and Saudi populations, which correlated with results obtained by comparison with autosomal profiles derived from the same individuals (Al-Snan et al. 2019).

Muharraq also fell closest to the only Bahraini data previously submitted to YHRD (YA004278, N = 156 Yfiler Plus profiles) although the sub-regional origins of the donors to that study are unknown.

## Discussion

While the addition of a number of RM Y-STRs greatly increased the discrimination capacity (DC) compared with the Yfiler multiplex previously implemented in Bahrain, a DC of 87.5% remains very low compared with most populations, for example Serbia 99.9% (Zgonjanin et al. 2017), Upper Austria and Salzburg 99.7% (Pickrahn et al. 2016), Mongolia 98.9% (Wang et al. 2019), Italy 98.5% (Rapone et al. 2016), US Caucasians 98.5% (Gopinath et al. 2016)**,** Daur 96.55% (Wang et al. 2019) and Saudi Arabia 95.3% (Khubrani et al. 2018), although the highly bottle-necked Greenland population is lower at 79.0% (Olofsson et al. 2015). There are many shared haplotypes in the Bahraini sample, despite precautions to exclude males sharing common patrilineal ancestry in the last three generations. This is likely the result of an extended period of rapid population expansion since the discovery of oil in the early 1930s. Increased prosperity since then has led to improvements in health care and a significantly reduced childhood mortality rate which, combined with a predominantly youthful population, has resulted in Bahrain having the tenth most rapidly growing population in the world (Al-Arrayed and Hamamy 2012).

Lying at the crossroads of Europe, Asia and Africa, the genetic landscape of Bahrain has been shaped by migrants from many other regions. Prior to the opening in 1986 of a 25-km causeway to Saudi Arabia (Fig. 1), all international contact had been via maritime routes through the Arabian Gulf. Unlike its immediate neighbors Saudi Arabia and Qatar, where haplogroup J1 predominates (71% and 58%, respectively (Cadenas et al. 2008; Iacovacci et al. 2017), the frequency of this haplogroup in Bahrain is just 23%, being highest in the largely desert Southern Governorate where ethnic Arabs are most common and declining in the more urban areas where the Persian Ajam and indigenous Baharna are most numerous. The diverse haplogroup composition hints at the variety of peoples who have left their mark on Bahrain with B2, E1b1a and E2 originating in Africa and H, L and R2 indicative of migration from South Asia, while the R1b haplotypes may result from the period of Portuguese rule from 1521 to 1602. We observed haplotypes predicted to belong to both primary branches of R1b, namely R1b1a-L754 (*n* = 10) and R1b1b-PH155 (*n* = 5): while R1b1a is by far the commonest worldwide and likely reflects European contact, the five examples predicted to belong to the very scarce basal haplogroup R1b1b-PH155 all carried a distinctive 25.1 intermediate allele at DYS481, showing that distinctive globally rare variants can locally reach high frequencies and emphasizing the relevance of appropriate regional databases.

In conclusion, we have characterized the paternal lineages of Bahrain, an island nation with a geographical and census size smaller than that of many modern cities. Based on the 27 Y-STRs in the Yfiler Plus multiplex kit, we observe a high and significant degree of differentiation between Bahrain’s four governorates (Northern, Southern, Muharraq, and Capital) which likely reflects differences in ethnic composition, in particular past contributions from the Arabian Peninsula, Africa and South Asia. We also observe an unusually high proportion of closely related haplotypes, the majority of which are found within, rather than between, governorates. Again, this reflects ethnic differences, exacerbated by the rapid population growth of the last 70 years. In the forensic context, our characterization of the high degree of population structure in Bahrain will aid in interpreting Y-STR evidence, and the much increased haplotype resolution offered by the Yfiler Plus kit compared to earlier multiplexes will be particularly valuable in improving discrimination power in this country.

## Electronic supplementary material

Below is the link to the electronic supplementary material.

Supplementary file1 (XLSX 121 kb)
